# Evidence quality assessment of traditional Chinese medicine and integrated traditional Chinese and Western medicine for acute upper respiratory infection in children: an umbrella review of systematic reviews and meta-analyses

**DOI:** 10.3389/fped.2026.1896116

**Published:** 2026-09-30

**Authors:** Zhen Liu, Yumeng Yang, Yuetong Liu, Yichen Lin, Zongkai Wang, Huiyang Shi, Zhenqi Wu

**Affiliations:** 1 School of Second Clinical Medicine, Liaoning University of Traditional Chinese, Medicine, Shenyang, China; 2 Dalian Medical University, Dalian, China; 3 Liaoning University of Traditional Chinese Medicine, Shenyang, China; 4 The Second Affiliated Hospital of Liaoning University of Traditional Chinese Medicine, Shenyang, China; 5 The Affiliated Hospital of Liaoning University of Traditional Chinese Medicine, Shenyang, China

**Keywords:** acute upper respiratory tract infection, children, integrated traditional Chinese and Western medicine, traditional Chinese medicine, umbrella review

## Abstract

**Objective:**

To systematically appraise the reporting completeness, methodological quality, risk of bias, and certainty of evidence for a prespecified outcome in systematic reviews and meta-analyses evaluating traditional Chinese medicine (TCM), either alone or in combination with conventional Western medicine, for acute upper respiratory tract infection (AURI) in children using an umbrella review approach.

**Methods:**

PubMed, Embase, Web of Science, the Cochrane Library, China National Knowledge Infrastructure (CNKI), Wanfang Data, VIP Database, and the Chinese Biomedical Literature Database (SinoMed) were systematically searched from database inception to February 5, 2026, without restrictions on publication year. Systematic reviews and meta-analyses evaluating TCM alone or TCM combined with conventional Western medicine for pediatric AURI were eligible for inclusion. Reporting completeness was assessed using the PRISMA 2020 statement, methodological quality using AMSTAR 2, and risk of bias using ROBIS. In addition, the certainty of evidence for the outcome of total effective rate was independently reassessed for each included systematic review using the GRADE framework.

**Results:**

A total of 41 systematic reviews and meta-analyses involving 57,590 children were included. The eligible reviews were published between 2010 and 2024; no systematic review or meta-analysis published between 2025 and February 5, 2026 met the prespecified eligibility criteria. Assessment against PRISMA 2020 identified varying degrees of underreporting, particularly with respect to protocol registration, complete search strategies, the influence of risk of bias on interpretation of findings, sensitivity analyses, and funding sources. According to AMSTAR 2, 37 reviews were rated as having moderate methodological quality, three as low quality, and one as critically low quality. ROBIS identified 38 reviews as being at high risk of bias. Across the 41 review-specific GRADE assessments of total effective rate, one was rated as moderate-certainty evidence, four as low-certainty evidence, and 36 as very-low-certainty evidence. These ratings apply exclusively to total effective rate, a non-standardized composite outcome, and should not be extrapolated to other outcomes such as time to fever resolution, duration of cough, or adverse events. Although most existing reviews suggested that TCM, either alone or combined with conventional Western medicine, may provide potential benefits in terms of total effective rate and selected short-term symptom outcomes, substantial clinical heterogeneity and methodological limitations were evident across the evidence base.

**Conclusion:**

Existing systematic reviews suggest that TCM, either alone or in combination with conventional Western medicine, may offer potential clinical benefits for children with AURI. However, the overall evidence base is characterized by a high risk of bias, and the certainty of evidence for total effective rate—a non-standardized composite outcome—is predominantly low or very low. The available evidence is therefore insufficient to support strong clinical recommendations, and the findings should be interpreted cautiously. Future studies should prioritize standardized, clinically meaningful, patient-relevant outcomes and strengthen the rigorous evaluation of both efficacy and safety.

## 1 Introduction

AURI is one of the most common infectious diseases in childhood and primarily affects the upper respiratory tract, including the nasal cavity, pharynx, and larynx (1). Because the immune system and respiratory defense mechanisms are not yet fully mature in children, they are particularly susceptible to viral and other respiratory pathogens. Common clinical manifestations include fever, nasal congestion, rhinorrhea, cough, sore throat, sputum production, and general malaise (2). AURI is characterized by a high incidence, acute onset, and rapid transmission and represents a major reason for pediatric outpatient and emergency department visits. Studies have shown that, in China, children with respiratory tract infections account for approximately 10%–20% of daily outpatient visits for respiratory diseases (3). Although most cases of pediatric AURI are self-limiting and predominantly viral in etiology, antiviral agents, antibiotics, and symptomatic treatments remain widely used in clinical practice. Inappropriate medication use may increase the risks of adverse drug reactions, antibiotic overuse, and antimicrobial resistance, thereby compromising medication safety and the quality of clinical management in children (4).

TCM and integrated traditional Chinese and Western medicine are widely used in the clinical management of pediatric AURI in China, commonly as adjunctive or combination therapies to alleviate symptoms such as fever, cough, nasal congestion, rhinorrhea, and sore throat (5). Various Chinese herbal formulations are used according to therapeutic principles such as clearing heat, relieving exterior syndromes, suppressing cough, and soothing the throat. Their potential therapeutic effects may involve antiviral and anti-inflammatory activities, immunomodulation, and attenuation of inflammatory responses in the respiratory mucosa (6). In recent years, an increasing number of randomized controlled trials have investigated TCM alone or in combination with conventional Western medicine for pediatric AURI, leading to the publication of multiple systematic reviews and meta-analyses and thereby expanding the evidence base in this field.

Systematic reviews and meta-analyses are important approaches for synthesizing evidence from clinical studies and play a key role in informing clinical decision-making and guideline development (7). To date, numerous systematic reviews and meta-analyses have reported that TCM or integrated traditional Chinese and Western medicine may offer advantages in improving the total effective rate, shortening the time to fever resolution, and relieving symptoms such as cough, sore throat, nasal congestion, and rhinorrhea in children with AURI. However, the reliability of conclusions drawn from systematic reviews depends not only on the quality of the included primary studies but also on the methodological rigor of the review itself, the completeness of reporting, the management of potential bias, and the certainty of the evidence (8). When the primary evidence is of low quality, outcome measures are inconsistently defined, or the review itself contains methodological limitations, statistically significant meta-analytic findings may still have limited clinical credibility.

The number of systematic reviews evaluating TCM and integrated traditional Chinese and Western medicine for pediatric AURI has continued to increase, yet substantial variation exists across reviews in terms of interventions, comparators, outcome measures, and approaches to quality assessment. In particular, composite and non-standardized outcomes such as the total effective rate are widely used, which may reduce the clinical interpretability and international comparability of the findings. Moreover, the overall quality of systematic reviews in this field has not been comprehensively characterized with respect to literature-search methods, protocol registration, management of risk of bias in primary studies, assessment of publication bias, and the certainty of evidence for specific outcomes. Therefore, the present study conducted an umbrella review of systematic reviews and meta-analyses evaluating TCM and integrated traditional Chinese and Western medicine for pediatric AURI. PRISMA 2020, AMSTAR 2, ROBIS, and GRADE were applied to comprehensively assess reporting completeness, methodological quality, risk of bias, and the certainty of evidence specifically for the outcome of total effective rate. This review aimed to provide a more objective appraisal of the credibility of the current evidence base and to inform the clinical application of TCM-related interventions for pediatric AURI, as well as the design and conduct of future high-quality clinical studies and systematic reviews.

## 2 Methods

### 2.1 Study design and registration

This study was conducted as an umbrella review to critically reassess systematic reviews and meta-analyses evaluating TCM, either alone or in combination with conventional Western medicine, for AURI in children. The protocol was prospectively registered with the International Prospective Register of Systematic Reviews (PROSPERO; registration No. CRD420261281709). This review was reported in accordance with the PRISMA 2020 statement (9).

### 2.2 Eligibility criteria

Systematic reviews or meta-analyses in which randomized controlled trials (RCTs) constituted the primary source of evidence were eligible for inclusion. Publications were restricted to Chinese or English. The study population comprised children with a clearly established diagnosis of AURI based on the eighth edition of Zhu Futang Practice of Pediatrics, the second edition of Pediatrics, or other relevant guidelines, textbooks, or clinical diagnostic criteria (10, 11). No restrictions were imposed on sex, geographic region, disease duration, or treatment course.

Eligible interventions included TCM administered alone or in combination with conventional Western medicine. No restrictions were imposed on the dosage form of TCM, which could include Chinese herbal injections, Chinese patent medicines, single-herb preparations, or compound herbal formulations. Control groups received conventional Western medical treatment, including antiviral agents, antibiotics, antipyretics, antitussives, expectorants, and other symptomatic or supportive treatments.

The primary outcome of interest was the total effective rate, as this was the most frequently reported outcome across the included systematic reviews. Other clinical outcomes, including time to fever resolution, time to resolution of pharyngeal symptoms, time to cough resolution, time to disappearance of herpetic lesions, and incidence of adverse reactions, were also extracted. Definitions of the total effective rate were primarily based on the response criteria used in the original studies and could vary across studies with respect to component events, response thresholds, and assessment time points. Accordingly, the clinical interpretation and certainty-of-evidence assessment of this outcome were undertaken cautiously in view of its non-standardized nature.

We excluded duplicate publications of the same systematic review; studies for which the full text was unavailable or key information could not be obtained; systematic reviews that did not include RCTs; network meta-analyses; narrative reviews; conference abstracts; dissertations and theses; study protocols; and publications in which the population, intervention, or outcomes were inconsistent with the scope of this review. When clearly overlapping versions addressing the same clinical question were published by the same research group, preference was given to the more recent publication, the version with more complete methodological information, or the study with more comprehensive data reporting.

### 2.3 Literature search strategy

A systematic electronic search was conducted in PubMed, Embase, Web of Science, the Cochrane Library, CNKI, Wanfang Data, the VIP Database, and the SinoMed. PROSPERO was additionally searched for relevant registered reviews. All databases were searched from inception to February 5, 2026, with no additional restriction on publication year.

The search strategy combined controlled vocabulary terms with free-text terms and was adapted to the characteristics of each database. The main English search terms included “Respiratory Tract Infections,” “Upper Respiratory Tract Infection,” “Common Cold,” “Tonsillitis,” “Pharyngitis,” “Herpangina,” “Chinese medicine,” “Traditional Chinese Medicine,” “child,” “children,” “systematic review,” and “Meta-analysis.” Chinese search terms included equivalents of “children,” “pediatric,” “infant,” “minor,” “acute upper respiratory tract infection,” “upper respiratory tract infection,” “common cold,” “acute tonsillitis,” “herpangina,” “acute nasopharyngitis,” “acute pharyngitis,” “traditional Chinese medicine,” “Chinese medicine,” “Chinese herbal medicine,” “integrated traditional Chinese and Western medicine,” “systematic review,” “meta-analysis,” and related terms.

### 2.4 Study selection and data extraction

All retrieved records were imported into EndNote for deduplication. Two reviewers independently screened the literature. Titles and abstracts were first reviewed to exclude clearly irrelevant studies, after which the full texts of potentially eligible publications were assessed against the predefined inclusion criteria. Any disagreement was resolved through discussion with a third reviewer.

Data were extracted independently by two reviewers using a prespecified data extraction form and subsequently cross-checked for accuracy. Extracted information included the first author, year of publication, number of primary studies included, sample size, intervention, comparator, treatment duration, outcome measures, effect estimates, heterogeneity statistics, methods used to assess risk of bias in the primary studies, and the main conclusions of each review.

For the GRADE reassessment, additional information specifically related to the total effective rate was extracted from each systematic review, including methodological limitations of the contributing primary studies, between-study heterogeneity, precision of effect estimates, directness of the populations and interventions, and assessment of publication bias. These data were used to independently reassess the certainty of evidence in the present umbrella review. Where available, GRADE ratings reported in the original systematic reviews were used only as supplementary information for cross-checking and were not directly adopted as the final GRADE judgments of this study. When information was incomplete, attempts were made to obtain additional data from the full text, supplementary materials, or, where feasible, by contacting the original authors.

### 2.5 Quality assessment

Two reviewers independently assessed the reporting completeness, methodological quality, risk of bias, and certainty of evidence of the included reviews. Disagreements were resolved through discussion, with arbitration by a third reviewer when necessary.

Reporting completeness was assessed using the PRISMA 2020 statement (9). PRISMA 2020 comprises 27 items. Based on the extent to which each item was reported, we classified reporting as “fully reported,” “partially reported,” or “not reported,” and descriptively summarized item-level reporting completeness. Because PRISMA 2020 is intended primarily to guide and assess the completeness of reporting in systematic reviews rather than to serve as a standardized numerical scoring instrument, no overall PRISMA 2020 score was calculated, and no arbitrary score thresholds were used to categorize reporting quality.

Methodological quality was assessed using AMSTAR 2 (12, 13). AMSTAR 2 consists of 16 items and classifies the overall methodological quality of systematic reviews and meta-analyses as high, moderate, low, or critically low according to weaknesses identified in critical and non-critical domains. Risk of bias was evaluated using the ROBIS tool (14, 15). The present study primarily applied Phases 2 and 3 of ROBIS. Phase 2 evaluates four domains: study eligibility criteria; identification and selection of studies; data collection and study appraisal; and synthesis and interpretation of findings. Phase 3 provides an overall judgment of risk of bias in the systematic review, categorized as low, high, or unclear.

The certainty of evidence was independently reassessed using the GRADE framework (16, 17). Under GRADE, the unit of assessment is a specific outcome together with its corresponding body of evidence; therefore, a single GRADE rating was not assigned to an entire systematic review. Because total effective rate was the most frequently reported outcome among the included reviews, GRADE reassessment was restricted to the body of evidence for this outcome within each systematic review. As the relevant evidence was derived predominantly from RCTs, the initial certainty was considered high and was subsequently downgraded, when appropriate, across five domains: methodological limitations, inconsistency, indirectness, imprecision, and publication bias. The final certainty of evidence was categorized as high, moderate, low, or very low. Other clinical outcomes, including time to fever resolution, duration of cough, and adverse events, were not subjected to GRADE reassessment. Accordingly, all GRADE ratings reported in this study apply exclusively to the outcome of total effective rate.

When an individual systematic review reported multiple intervention regimens, subgroup analyses, or more than one meta-analysis for total effective rate, these effect estimates were considered components of the same outcome-specific body of evidence within that review and were not assigned separate GRADE ratings. Instead, a single GRADE judgment was made for the body of evidence on total effective rate in each systematic review by jointly considering risk of bias in the contributing primary studies, inconsistency, indirectness, imprecision, and publication bias. Thus, the present study generated review-specific certainty ratings for total effective rate rather than pooled GRADE ratings across multiple clinical outcomes within the same review.

The same primary RCTs may have been included in more than one systematic review. Because a complete citation matrix linking individual RCTs to the included systematic reviews was not constructed, quantitative adjustment for study overlap was not feasible. Consequently, GRADE ratings from different systematic reviews were not further combined into a single cross-review estimate of overall certainty, nor was the overall strength of evidence inferred from the number of reviews falling within a particular GRADE category. The review-specific GRADE judgments may therefore have been based on partially overlapping primary evidence and should not be interpreted as representing 41 fully independent bodies of evidence.

In addition, definitions of total effective rate varied across primary studies with respect to component events, response thresholds, and assessment time points. The corresponding GRADE findings should therefore be interpreted cautiously in light of the non-standardized nature of this composite outcome. Because definitions, monitoring procedures, and follow-up durations for adverse events also varied considerably across systematic reviews, safety outcomes were not independently assessed using GRADE. Accordingly, the GRADE ratings presented in this study should not be extrapolated to adverse events or other safety outcomes.

### 2.6 Data analysis

Descriptive analyses were used to summarize the basic characteristics of the included systematic reviews and meta-analyses, including interventions, outcomes, reporting completeness, methodological quality, risk of bias, and certainty of evidence. Categorical variables were summarized as frequencies and percentages, whereas continuous variables were described using means, ranges, or medians, as appropriate to the distribution and availability of the data. Because the primary purpose of this umbrella review was to evaluate the reporting completeness, methodological quality, risk of bias, and certainty of evidence for a prespecified outcome in existing systematic reviews, no re-analysis or re-pooling of data from the original RCTs was undertaken, and no second-order meta-analysis of effect estimates across systematic reviews was performed.

Potential overlap of primary RCTs across systematic reviews and meta-analyses was considered an important methodological limitation when interpreting the findings. Because a complete one-to-one citation matrix linking all primary RCTs to each included systematic review had not been prespecified or constructed, quantitative overlap measures such as the CCA were not calculated. To minimize the influence of overlapping evidence on interpretation, neither the number of positive systematic reviews nor the apparent consistency of conclusions across reviews was treated as an independent indicator of evidence strength. Instead, the overall evidence base was interpreted cautiously with primary consideration given to the findings of AMSTAR 2, ROBIS, and GRADE.

## 3 Results

### 3.1 Study selection and characteristics of the included reviews

The initial search identified 692 potentially relevant records. After removal of duplicates using EndNote, screening of titles and abstracts, and full-text assessment, 41 systematic reviews and meta-analyses were ultimately included. The study selection process is presented in Figure 1. The literature search was conducted up to February 5, 2026, with no restriction on publication year. After application of the prespecified eligibility criteria, no systematic review or meta-analysis published between 2025 and February 5, 2026 met the inclusion criteria; therefore, the included reviews were published between 2010 and 2024. Of these, 38 were published in Chinese and three in English, comprising a total of 57,590 participants (18–58).

**Figure 1:**
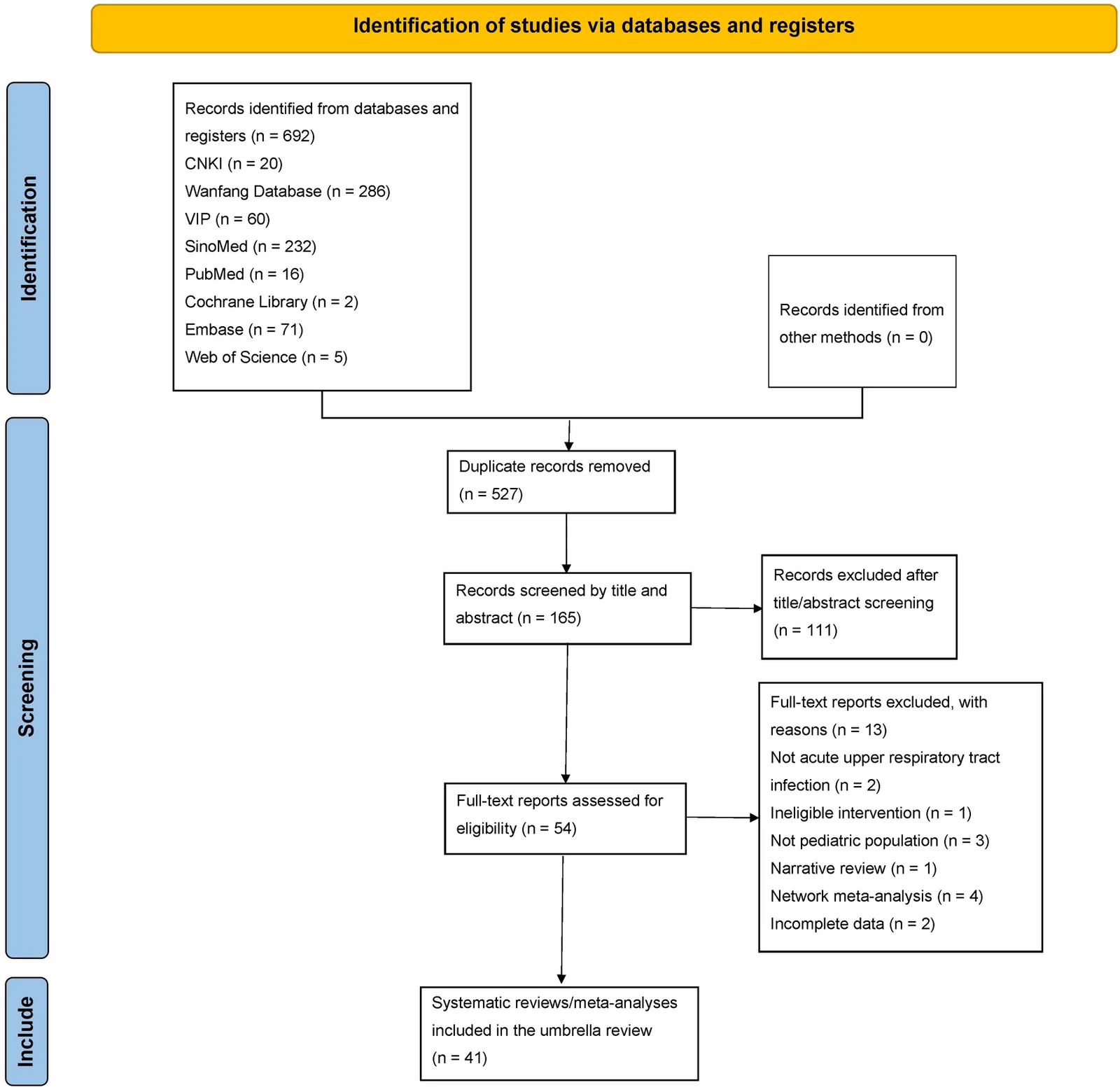
PRISMA flow diagram.

The included reviews evaluated a wide range of TCM preparations, including Reduning Injection, Xiyanping Injection, Yanhuning Injection, Tanreqing Injection, Qingkailing Injection, Xiaoer Chiqiao Qingre Granules, Lanqin Oral Liquid, Pudilan Xiaoyan Oral Liquid, Shufeng Jiedu Capsules, and Xiaoer Chaigui Tuire Granules. According to intervention type, 14 reviews evaluated TCM alone, 11 evaluated integrated traditional Chinese and Western medicine, and 16 included both TCM monotherapy and combination regimens. Control groups mainly received conventional antiviral therapy, antibiotics, and symptomatic or supportive treatment. Among studies that explicitly reported treatment duration, treatment courses generally ranged from 3 to 14 days.

With respect to assessment of the methodological quality of the primary studies, 25 reviews used the Cochrane risk-of-bias tool, 13 used the Jadad scale, and three applied both instruments. The basic characteristics of the included reviews are summarized in Table 1.

**Table 1:** Characteristics of included reviews.

Category	Form	Included Review	No. of studies/sample size	Intervention	Control	Duration (days)	Outcomes	Quality assessment tool
TCM monotherapy	Injection	Li 2010	11/1209	RDN	RBV	3	1,2,3	Jadad Scale
TCM monotherapy	Injection	Wu 2013	12/1215	YHN	RBV	3–7	1,2,4,5,6	Jadad Scale
TCM monotherapy	Injection	Shi 2014	7/857	RDN	RBV	3–7	1,6	Jadad Scale
TCM monotherapy	Injection	Tao 2015	7/1055	RDN	RBV	3–7	1,5,6	Jadad Scale
TCM monotherapy	Injection	Guo 2016	6/978	RDN	RBV	NA	1,2,16,3,10,11,5	Jadad Scale
TCM monotherapy	Injection	Han 2016	12/1653	TRQ	RBV	3–7	1,2,5,11,12,16	Cochrane Risk of Bias Tool + Jadad Scale
TCM monotherapy	Injection	Lian 2016	14/1939	XYP	RBV	NA	1,2,4	Jadad Scale
TCM monotherapy	Injection	Liu 2017	10/1486	XYP	RBV	NA	1,2,16,3,5,10	Cochrane Risk of Bias Tool
TCM monotherapy	Injection	Liu 2017	17/1750	XYP, TRQ	RBV	NA	1,2,5,16	Cochrane Risk of Bias Tool
TCM monotherapy	Injection	Ren 2017	15/2457	XYP	RBV	NA	1,2,16,11	Cochrane Risk of Bias Tool
TCM monotherapy	Oral preparation	Zhang 2015	9/1232	LQ	RBV	3–7	1	Cochrane Risk of Bias Tool
TCM monotherapy	Oral preparation	Yuan 2016	15/1916	CEQR	RBV	3–5	1,2,3,10,7,8,16	Cochrane Risk of Bias Tool
TCM monotherapy	Oral preparation	Zhong 2017	7/782	JLQR	RBV	3–5	1,2,3,16,5,11	Jadad Scale
TCM monotherapy	Oral preparation	Hu 2017	33/7175	Andro	CWAT	3–7	1,6	Cochrane Risk of Bias Tool
TCM monotherapy	Oral preparation	Ma 2020	10/1250	CEQR	RBV	3–5	1,2,3,16,5	Cochrane Risk of Bias Tool
Integrative therapy	Injection	Fang 2012	13/1376	RDN + RBV	RBV	3–7	1,2,4,6	Jadad Scale
Integrative therapy	Injection	Xu 2016	23/2796	XYP + RBV	RBV	3	1,2,4,5	Cochrane Risk of Bias Tool + Jadad Scale
Integrative therapy	Injection	Li 2016	22/3788	QKL + CWAT	CWAT	3–5	1,2,5	Cochrane Risk of Bias Tool
Integrative therapy	Injection	Peng 2019	10/1104	YHN + IFN-α	IFN-α	4–5	1,2,16,10	Cochrane Risk of Bias Tool
Integrative therapy	Oral preparation	Luo 2011	2/500	GKSQ + CWAT	CWAT	NA	1	Cochrane Risk of Bias Tool
Integrative therapy	Oral preparation	Chen 2013	12/1696	CEQR + CWAT	CWAT	3–6	1,2,4,6	Jadad Scale
Integrative therapy	Oral preparation	Tao 2017	24/2788	KHJ + CWAT	CWAT	3–7	1,2,3,4,5	Jadad Scale
Integrative therapy	Oral preparation	Rong 2021	12/1254	AEN + CWAT	CWAT	3–7	1,2,3,16	Cochrane Risk of Bias Tool
Integrative therapy	Oral preparation	Zhang 2021	25/3410	SFJD + CWAT	CWAT	5–7	2,3,4,6,16	Cochrane Risk of Bias Tool
Integrative therapy	Oral preparation	Wang 2023	16/1754	SFJD + CWAT	CWAT	3–14	1,2,3,5,9,10,15	Cochrane Risk of Bias Tool
Mixed interventions	Injection	Chen 2012	15/2457	XYP/XYP + CWAT	CWAT	3–7	1,2,4,5	Cochrane Risk of Bias Tool
Mixed interventions	Injection	Guo 2016	37/7232	YHN/YHN + CWAT	CWAT	NA	1,2,3,5,10,11,16	Jadad Scale
Mixed interventions	Oral preparation	Qiu 2014	14/1989	SSP/QJT/PSDAS/HQTR/JWSJ/RDQ/SNS/XYP/CGTR + CWAT	CWAT/RBV/CXM	3–6	1	Jadad Scale
Mixed interventions	Oral preparation	Duan 2016	6/763	LQ/LQ + RBV/LQ + CWAT	CWAT/RBV	NA	1	Cochrane Risk of Bias Tool + Jadad Scale
Mixed interventions	Oral preparation	Liu 2016	17/1740	YQ/QJLY/QYJD + RBV/NHTMLY + CWAT/XXDC + RBV/QJXH + RBV/QQYQ + RBV/JDQH + CWAT	CWAT/RBV	3–7	1,2,4,5	Jadad Scale
Mixed interventions	Oral preparation	Chen 2018	14/2171	CGTR/CGTR + RBV	RBV	3–7	1,2,10	Cochrane Risk of Bias Tool
Mixed interventions	Oral preparation	Wang 2019	26/2752	SHL/SHL + RBV/SHL + CWAT/SHL + PDT	RBV/CWAT/PDT/QKL	2–7	1,2,3,5,16	Cochrane Risk of Bias Tool
Mixed interventions	Oral preparation	Yuan 2019	11/1204	LQ/LQ + RBV	RBV	5	1,2,4,5	Cochrane Risk of Bias Tool
Mixed interventions	Oral preparation	Bai 2020	16/1924	PDL + CXM/PDL + CWAT/PDL + CEQR	CWAT/CXM/RBV	NA	1,2,3,5,7,8,10,16	Cochrane Risk of Bias Tool
Mixed interventions	Oral preparation	Xu 2020	10/1288	PDL/PDL + ACV	ACV	NA	1,2,4	Jadad Scale
Mixed interventions	Oral preparation	Cai 2022	13/1980	JEQJ/JEQJ + CWAT	CWAT	5–7	1,2,3,4,5,9,11,16	Cochrane Risk of Bias Tool
Mixed interventions	Oral preparation	Zhu 2022	3/834	QXQJ/QXQJ + CWAT	CWAT	3	1,5	Cochrane Risk of Bias Tool
Mixed interventions	Oral preparation	Shi 2023	11/1015	YQ/YQ + CWAT	CWAT	3–7	1,2,5	Cochrane Risk of Bias Tool
Mixed interventions	Oral preparation	Zhong 2023	18/2459	CH/CH + CWAT	CWAT	5–10	1,2,3,16,5,7,8,13,14	Cochrane Risk of Bias Tool
Mixed interventions	Oral preparation	Li 2024	20/2913	CGTR/CGTR + RBV	RBV/CWAT	3–12	1,2,3,6,9,10,11,16	Cochrane Risk of Bias Tool
Mixed interventions	Oral preparation	Xu 2024	16/2287	KG/KG + CWAT/KG + PDT	CWAT/PDT	2–14	1,2,5,16	Cochrane Risk of Bias Tool

TCM, traditional Chinese medicine; NA, not available.

Interventions: RDN, Reduning Injection; XYP, Xiyanping Injection; YHN, Yanhuning Injection; TRQ, Tanreqing Injection; QKL, Qingkailing Injection; GKSQ, Ganke Shuangqing Capsules; CEQR, Xiaoer Chiqiao Qingre Granules; SHL, Shuanghuanglian Oral Liquid/Granules; LQ, Lanqin Oral Liquid; CGTR, Xiaoer Chaigui Tuire Granules; PDL, Pudilan Xiaoyan Oral Liquid; SFJD, Shufeng Jiedu Capsules; JLQR, Jinlian Qingre Effervescent Tablets; Andro, Andrographis paniculata; KHJ, Kaihoujian Spray; AEN, An’erning Granules; JEQJ, Jian’er Qingjie Liquid; QXQJ, Qinxiang Qingjie Oral Liquid; YQ, Yinqiao Powder; CH, Chaihuang Granules; KG, Kanggan Granules; QJLY, Qingjie Liyan Decoction; QYJD, Qingyan Jiedu Decoction; NHTMLY, Niuhuang Tianma Liyan Powder; XXDC, Xiexin Daochi Decoction; QJXH, Qingjie Xiehuang Decoction; QQYQ, Qingqin Yinqiao Decoction; JDQH, Jiedu Qinghuo Decoction; SSP, Shujie Powder; QJT, Qingjiang Tablets; PSDAS, Potassium Sodium Dehydroandrographolide Succinate; HQTR, Hanqing Tuire Decoction; JWSJ, Jiuwei Shuangjie Oral Liquid; RDQ, Reduqing Oral Liquid; SNS, Sini Powder.

Controls: CWAT, conventional Western antiviral therapy; RBV, ribavirin; IFN-α, interferon-α; PDT, pidotimod; ACV, acyclovir; CXM, cefuroxime sodium.

Outcomes: 1, overall clinical response rate; 2, time to fever resolution; 3, time to sore throat resolution; 4, time to herpes resolution; 5, incidence of adverse events; 6, cure rate; 7, tumor necrosis factor-α; 8, interleukin-6; 9, time to pharyngeal swelling resolution; 10, time to resolution of nasal congestion and rhinorrhea; 11, time to resolution of pharyngeal hyperemia; 12, improvement in tonsillar enlargement; 13, cellular immune function (CD3+, CD4+, CD8+, CD4+/CD8+); 14, humoral immune function (IgA, IgM, IgG); 15, disease recurrence frequency; 16, time to cough resolution.

### 3.2 PRISMA 2020 assessment

Item-level assessment using PRISMA 2020 revealed variability in reporting completeness across the 41 included systematic reviews and meta-analyses. Overall, most reviews adequately reported basic information, including the study objectives, eligibility criteria, characteristics of the included studies, and main findings. However, varying degrees of underreporting were identified in several areas, particularly protocol registration, presentation of complete search strategies, consideration of how risk of bias in the primary studies affected interpretation of the findings, sensitivity analyses, and reporting of funding sources. The distribution of items classified as fully reported, partially reported, or not reported across the PRISMA 2020 checklist is presented in Figure 2.

**Figure 2:**
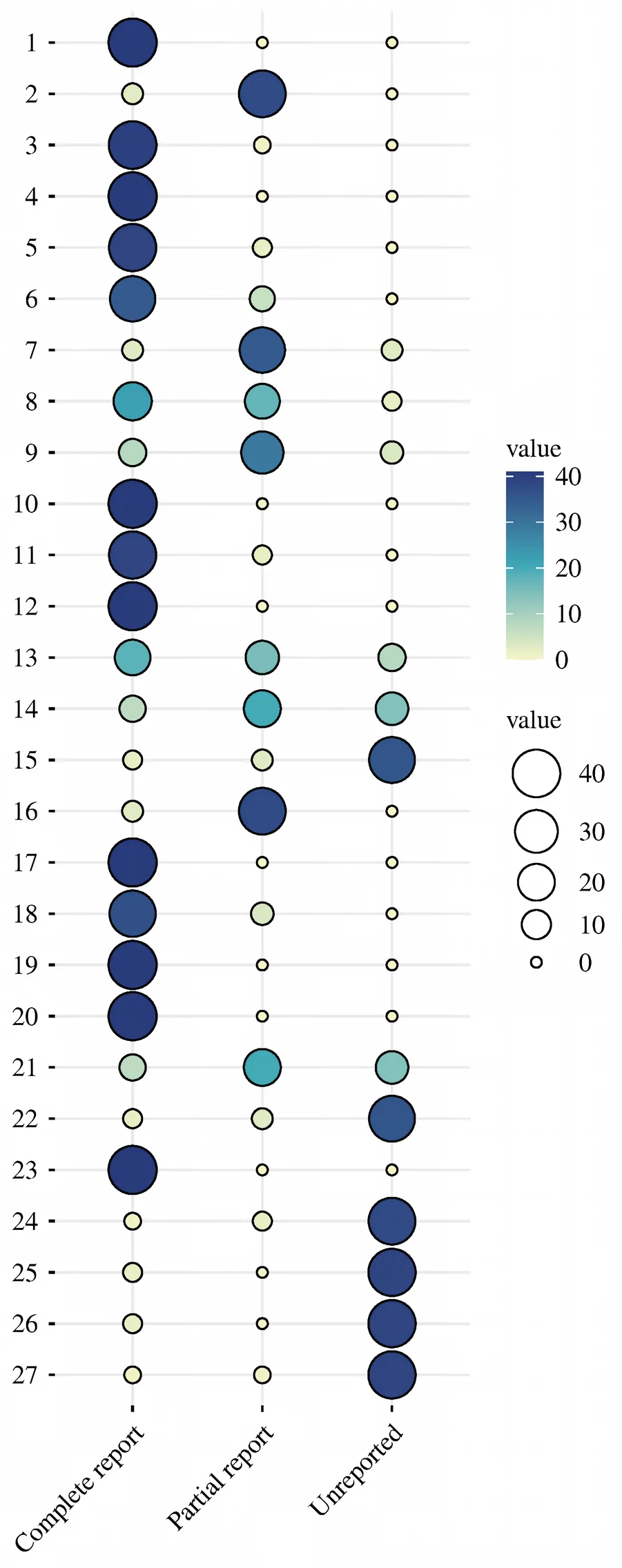
PRISMA2020 assessment.

### 3.3 AMSTAR 2 assessment

The AMSTAR 2 assessment indicated that the methodological quality of the included systematic reviews and meta-analyses was predominantly moderate. Among the 41 included reviews, 37 were rated as having moderate methodological quality, three as low quality, and one as critically low quality; none was rated as high quality. At the item level, deficiencies were identified in some reviews for Item 13, which assesses whether the risk of bias in the included primary studies was considered when interpreting or discussing the results, and Item 15, which assesses whether publication bias was adequately investigated and its potential impact discussed. These findings indicate that the handling of risk of bias in primary studies and the assessment of publication bias remain areas requiring further improvement. Among the non-critical items, reporting of funding sources and potential conflicts of interest of the primary studies was also insufficient.

It should be noted that AMSTAR 2 primarily evaluates the methodological quality and overall confidence in the results of a systematic review, whereas ROBIS is designed to assess the risk of bias that may arise during the conduct of the review. Therefore, the two instruments differ in their objectives and judgment frameworks. The AMSTAR 2 and ROBIS findings should consequently be regarded as complementary assessments of different dimensions of the existing evidence base. Their relationship is discussed further in the Discussion section. The results of the AMSTAR 2 assessment are presented in Figure 3.

**Figure 3:**
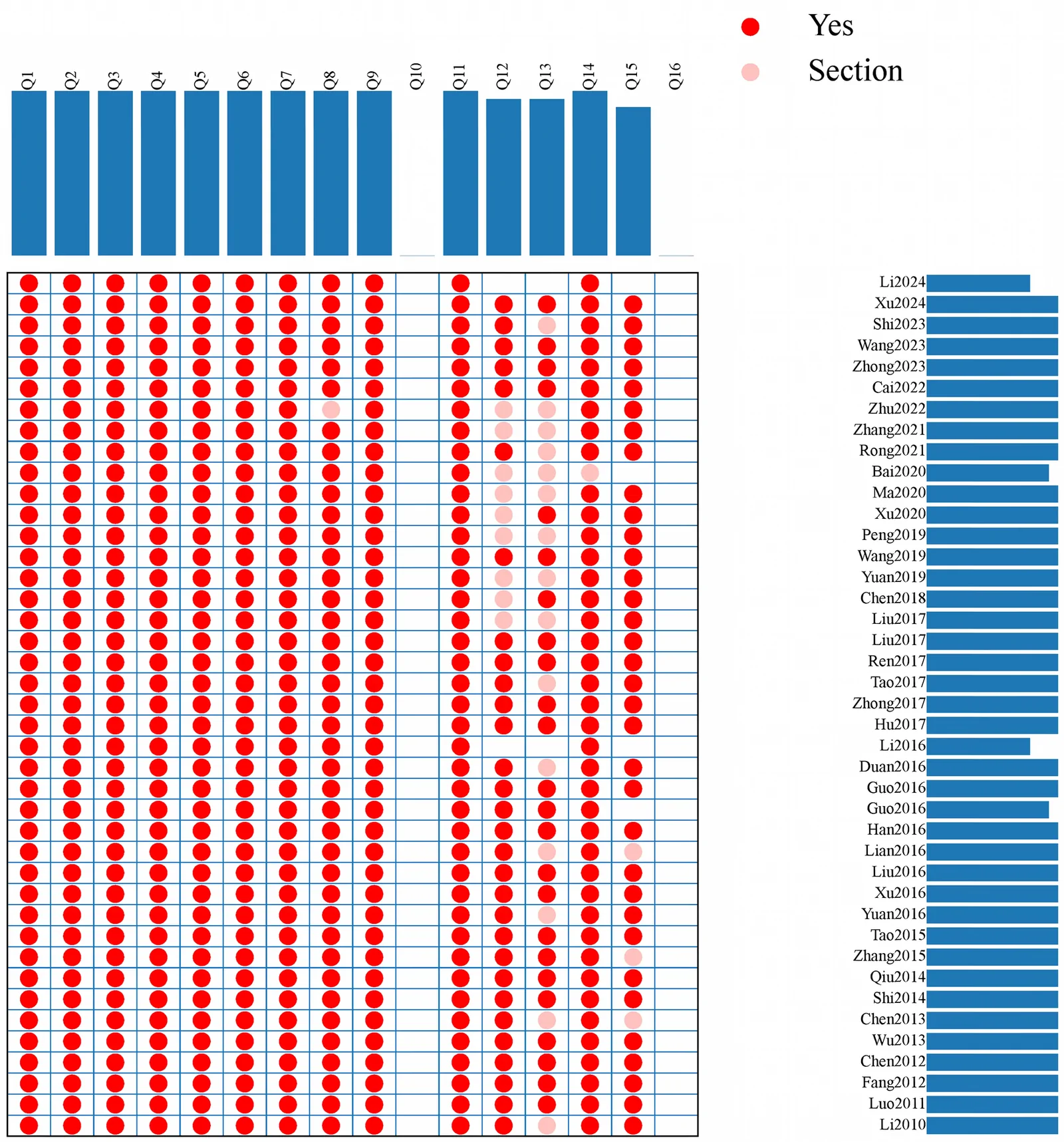
AMSTAR2 assessment.

### 3.4 ROBIS assessment

The ROBIS assessment indicated that the overall risk of bias among the included systematic reviews and meta-analyses was high (Figure 4). Across the domains of study eligibility criteria, identification and selection of studies, data collection and study appraisal, and synthesis and interpretation of findings, most reviews exhibited varying degrees of concern regarding potential bias. Overall, only one review was judged to be at low risk of bias, two were judged to have some concerns, and the remaining 38 were rated as being at high risk of bias.

**Figure 4:**
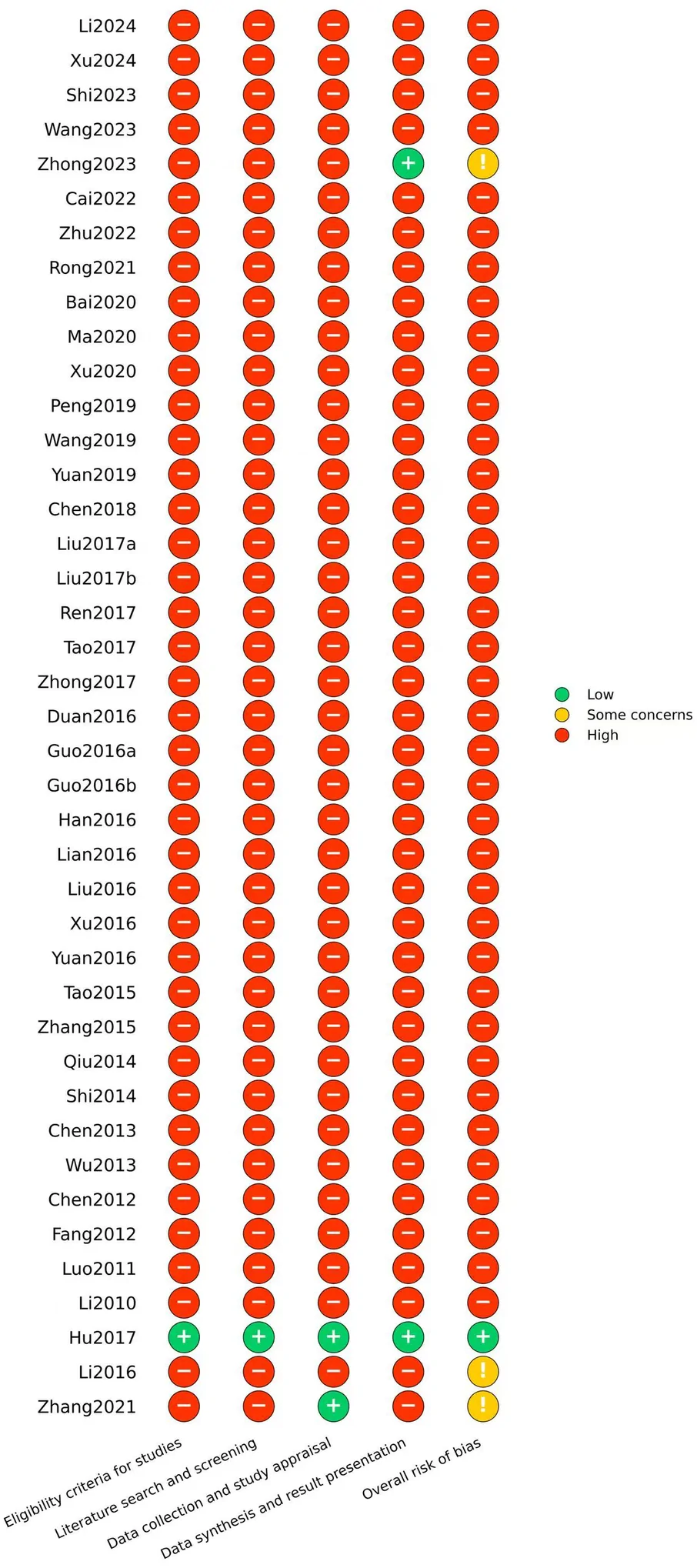
ROBIS assessment.

Specifically, the review by Hu et al. was judged to be at low risk of bias across all assessed domains. The reviews by Zhong et al. and Zhang et al. performed comparatively well in several domains but nevertheless raised some concerns overall. These findings suggest that the current body of systematic review evidence may be affected by methodological limitations in study identification and selection, appraisal of primary studies, and synthesis and interpretation of findings. Consequently, the overall credibility of the existing evidence base remains limited.

### 3.5 GRADE assessment

The GRADE framework was used to independently assess the certainty of the body of evidence specifically for the outcome of total effective rate within each included systematic review or meta-analysis; no single GRADE rating was assigned to an entire review. Taking the body of evidence for total effective rate within each systematic review as one assessment unit, a total of 41 review-specific GRADE judgments were generated. Of these, one was rated as moderate-certainty evidence, four as low-certainty evidence, and 36 as very-low-certainty evidence.

The main reasons for downgrading the certainty of evidence included methodological limitations of the primary studies, inconsistency across studies, imprecision, and potential publication bias. Inadequate reporting of random sequence generation, allocation concealment, and blinding was among the most common methodological concerns. These GRADE ratings apply exclusively to the body of evidence for total effective rate within each systematic review. They do not imply that other clinical outcomes within the same review, such as time to fever resolution, duration of cough, or adverse events, have the same certainty of evidence, nor should they be interpreted as an overall certainty rating for the systematic review as a whole.

It should also be emphasized that the same primary RCTs may have been included in multiple systematic reviews. Consequently, the 41 GRADE judgments may have been based on partially overlapping primary evidence and should not be regarded as 41 completely independent bodies of evidence. Accordingly, these GRADE ratings were not further pooled to generate a single cross-review estimate of overall certainty, and the overall strength of evidence was not inferred from the number of systematic reviews assigned to any particular GRADE category.

Furthermore, total effective rate was generally defined by the primary studies themselves and typically represented a composite of response categories such as “cured,” “markedly effective,” and “effective.” The component events, response thresholds, and assessment time points were not fully consistent across studies. Therefore, even when individual meta-analyses demonstrated statistically significant benefits, the clinical interpretability, reproducibility, and international comparability of this outcome remain limited. The results of the GRADE assessment are presented in Table 2.

**Table 2:** GRADE assessment.

Outcome	Included review	Intervention	No. of studies	Sample size: intervention	Sample size: control	Effect estimate	I^2^ (%)	Risk of bias	Inconsistency	Indirectness	Imprecision	Publication bias	Certainty of evidence
Overall clinical response rate	Li 2010	RDN	2	135	92	OR=4.25	NA	−2	0	−1	−1	−1	Very low
Overall clinical response rate	Wu 2013	YHN	12	621	594	RR = 4.82	0	−2	0	−1	0	−1	Very low
Overall clinical response rate	Shi 2014	XYP	7	437	420	OR=3.54	53	−2	−1	−1	0	−1	Very low
Overall clinical response rate	Tao 2015	RDN	7	540	515	RR = 1.76	0	−2	0	−1	−1	0	Very low
Overall clinical response rate	Guo 2016	RDN	6	494	484	OR=3.42	0	−1	0	−1	0	0	Low
Overall clinical response rate	Han 2016	TRQ	12	910	743	OR=6.42	0	−2	0	−1	0	−1	Very low
Overall clinical response rate	Lian 2016	XYP	13	943	920	OR=4.69	30	−2	0	−1	0	−1	Very low
Overall clinical response rate	Liu 2017	XYP	17	888	887	OR=4.87	0	−1	0	−1	0	0	Low
Overall clinical response rate	Liu 2017	XYP, TRQ	15	1258	1199	OR=5.88	28	−2	0	−1	0	−1	Very low
Overall clinical response rate	Ren 2017	XYP	9	700	700	RR = 1.19	62	−2	−1	−1	0	0	Very low
Overall clinical response rate	Zhang 2015	LQ	9	642	590	RR = 1.27	36	−2	0	−1	0	−1	Very low
Overall clinical response rate	Yuan 2016	CEQR	15	1011	905	OR=2.90	50	−1	0	−1	0	−1	Very low
Overall clinical response rate	Zhong 2017	JLQR	7	392	390	RR = 1.20	0	−2	0	−1	−1	−1	Very low
Overall clinical response rate	Hu 2017	Andro	33	1207	1096	RR = 1.44	89	−1	−2	−1	−1	0	Very low
Overall clinical response rate	Ma 2020	CEQR	10	667	583	RR = 1.25	33	−2	0	−1	0	−1	Very low
Overall clinical response rate	Fang 2012	RDN + RBV	12	630	626	OR=3.40	0	−2	0	−1	0	−1	Very low
Overall clinical response rate	Xu 2016	XYP + RBV	23	1427	1369	OR=4.53	0	−2	0	−1	0	−1	Very low
Overall clinical response rate	Li 2016	QKL + CWAT	14	1142	1096	RR = 1.18	0	−1	0	−1	0	0	Low
Overall clinical response rate	Peng 2019	YHN + IFN-α	10	549	555	OR=4.99	0	−2	0	−1	0	−1	Very low
Overall clinical response rate	Luo 2011	GKSQ + CWAT	2	253	247	RR = 1.15	0	−1	0	−1	−1	0	Very low
Overall clinical response rate	Chen 2013	CEQR + CWAT	12	809	800	RR = 1.19	14	−1	0	−1	0	−1	Very low
Overall clinical response rate	Tao 2017	KHJ + CWAT	24	1860	1728	OR=4.92	4	0	0	−1	0	0	Moderate
Overall clinical response rate	Rong 2021	AEN + CWAT	12	878	663	OR=4.08	0	−1	0	−1	0	−1	Very low
Overall clinical response rate	Zhang 2021	SFJD + CWAT	8	570	556	RR = 1.22	17	−2	0	−1	−1	0	Very low
Overall clinical response rate	Wang 2023	SFJD + CWAT	16	893	861	RR = 1.14	0	−1	0	−1	0	−1	Very low
Overall clinical response rate	Chen 2012	XYP/XYP + CWAT	15	2457	1258	RR = 1.22	29	−2	0	−1	0	−1	Very low
Overall clinical response rate	Guo 2016	YHN/YHN + CWAT	37	3439	3283	OR=3.98	11	0	−1	0	0	−2	Low
Overall clinical response rate	Qiu 2014	SSP/QJT/PSDAS/HQTR/JWSJ/RDQ/SNS/XYP/CGTR + CWAT	14	1039	950	OR=3.66	NA	−2	0	−1	0	−1	Very low
Overall clinical response rate	Duan 2016	LQ/LQ + RBV/LQ + CWAT	6	396	367	OR=3.46	0	−2	0	−1	0	−1	Very low
Overall clinical response rate	Liu 2016	YQ/QJLY/QYJD + RBV/NHTMLY + CWAT/XXDC + RBV/QJXH + RBV/QQYQ + RBV/JDQH + CWAT	17	929	811	OR=4.80	0	−2	0	−1	0	−1	Very low
Overall clinical response rate	Chen 2018	CGTR/CGTR + RBV	14	1072	1049	OR=5.43/OR=0.70	0/67	−1	−1	−1	0	0	Very low

GRADE, Grading of Recommendations Assessment, Development and Evaluation; OR, odds ratio; RR, risk ratio; NA, not available. Negative values indicate downgrading levels for each GRADE domain. RDN, Reduning Injection; YHN, Yanhuning Injection; XYP, Xiyanping Injection; TRQ, Tanreqing Injection; LQ, Lanqin Oral Liquid; CEQR, Xiaoer Chiqiao Qingre Granules; JLQR, Jinlian Qingre Effervescent Tablets; Andro, Andrographis paniculata; RBV, ribavirin; QKL, Qingkailing Injection; CWAT, conventional Western antiviral therapy; IFN-α, interferon-α; GKSQ, Ganke Shuangqing Capsules; KHJ, Kaihoujian Spray; AEN, An’erning Granules; SFJD, Shufeng Jiedu Capsules; SSP, Shujie Powder; QJT, Qingjiang Tablets; PSDAS, Potassium Sodium Dehydroandrographolide Succinate; HQTR, Hanqing Tuire Decoction; JWSJ, Jiuwei Shuangjie Oral Liquid; RDQ, Reduqing Oral Liquid; SNS, Sini Powder; CGTR, Xiaoer Chaigui Tuire Granules; YQ, Yinqiao Powder; QJLY, Qingjie Liyan Decoction; QYJD, Qingyan Jiedu Decoction; NHTMLY, Niuhuang Tianma Liyan Powder; XXDC, Xiexin Daochi Decoction; QJXH, Qingjie Xiehuang Decoction; QQYQ, Qingqin Yinqiao Decoction; JDQH, Jiedu Qinghuo Decoction.

### 3.6 Distribution of outcome measures and summary of efficacy and safety findings

The outcomes reported across the included reviews primarily comprised total effective rate, duration or time to resolution of clinical symptoms, inflammatory markers, immune function indicators, and incidence of adverse events. Total effective rate was the most frequently reported outcome and was assessed in the vast majority of the included reviews. Symptom-related outcomes mainly included time to fever resolution, time to cough resolution, time to disappearance of sore throat, time to relief of nasal congestion and rhinorrhea, time to resolution of herpetic lesions, time to disappearance of pharyngeal congestion, and improvement in tonsil-related symptoms. In contrast, inflammatory and immune-related outcomes were reported less frequently, with only a subset of reviews evaluating markers such as IL-6, TNF-α, CD3⁺, CD4⁺, IgA, IgM, and IgG. The distribution of outcomes across the included reviews is presented in Table 3.

**Table 3:** Endpoint indicator distribution.

Category	Form	Included review	Total effective rate	Clinlcal symptom														Inflammatory factor			Immunologicalarkers	ADR
				T	C	R	N	H	P	G	L	S	Y	O	F	D	K	IL-2	IL-6	TNF-α		
TCM monotherapy	Injection	Li 2010	+	+	/	/	/	/	+	/	/	/	/	/	/	/	/	/	/	/	/	/
TCM monotherapy	Injection	Wu 2013	+	+	/	/	/	+	/	/	/	/	/	/	/	/	/	/	/	/	/	+
TCM monotherapy	Injection	Shi 2014	+	/	/	/	/	/	/	/	/	/	/	/	/	/	/	/	/	/	/	/
TCM monotherapy	Injection	Tao 2015	+	/	/	/	/	/	/	/	/	/	/	/	/	/	/	/	/	/	/	+
TCM monotherapy	Injection	Guo 2016	+	+	+	+	+	/	/	/	/	/	+	/	/	/	/	/	/	/	/	+
TCM monotherapy	Injection	Han 2016	+	/	+	/	/	/	/	/	/	/	+	+	+	/	/	/	/	/	/	+
TCM monotherapy	Injection	Lian 2016	+	+	/	/	/	+	/	/	/	+	/	/	/	/	/	/	/	/	/	+
TCM monotherapy	Injection	Liu 2017	+	+	+	+	+	/	+	/	/	/	/	/	/	/	/	/	/	/	/	+
TCM monotherapy	Oral preparation	Zhang 2015	+	/	/	/	/	/	/	/	/	/	/	/	/	/	/	/	/	/	/	/
TCM monotherapy	Oral preparation	Yuan 2016	+	+	+	+	+	/	+	/	/	/	/	/	/	/	/	/	+	+	/	/
TCM monotherapy	Oral preparation	Zhong 2017	+	/	+	/	/	/	+	/	/	/	+	/	/	/	/	/	/	/	/	+
TCM monotherapy	Oral preparation	Hu 2017	+	/	/	/	/	/	/	/	/	/	/	/	/	/	/	/	/	/	/	/
TCM monotherapy	Oral preparation	Ma 2020	+	+	+	/	/	/	+	/	/	/	/	/	/	/	/	/	/	/	/	+
Integrative therapy	Injection	Fang 2012	+	+	/	/	/	+	/	/	/	/	/	/	/	/	/	/	/	/	/	/
Integrative therapy	Injection	Xu 2016	+	+	/	/	/	+	/	/	/	/	/	/	/	/	/	/	/	/	/	+
Integrative therapy	Injection	Li 2016	+	/	/	/	/	/	/	/	/	/	/	/	+	/	/	/	/	/	/	+
Integrative therapy	Injection	Peng 2019	+	+	+	+	+	/	/	/	/	/	/	/	/	/	/	/	/	/	/	/
Integrative therapy	Oral preparation	Luo 2011	+	/	/	/	/	/	/	/	/	/	/	/	/	/	/	/	/	/	/	/
Integrative therapy	Oral preparation	Chen 2013	+	+	+	+	+	/	+	/	/	/	/	/	/	/	/	/	+	+	/	+
Integrative therapy	Oral preparation	Tao 2017	+	/	/	/	/	+	+	/	/	+	/	/	+	/	/	/	/	/	/	+
Integrative therapy	Oral preparation	Rong 2021	+	+	+	/	/	/	+	+	+	/	/	/	/	/	/	/	/	/	/	/
Integrative therapy	Oral preparation	Zhang 2021	+	+	+	/	/	+	+	/	/	/	/	/	/	/	/	/	/	/	/	/
Integrative therapy	Oral preparation	Wang 2023	+	+	+	+	+	/	+	/	/	/	/	/	/	/	/	/	/	/	/	+
Mixed interventions	Injection	Chen 2012	+	+	/	/	/	+	/	/	/	/	/	/	/	/	/	/	/	/	/	+
Mixed interventions	Injection	Guo 2016	+	+	+	+	+	/	+	/	/		+	/	/	/	/	/	/	/	/	+
Mixed interventions	Oral preparation	Qiu 2014	+	/	/	/	/	/	/	/	/	/	/	/	/	/	/	/	/	/	/	/
Mixed interventions	Oral preparation	Duan 2016	+	/	/	/	/	/	/	/	/	/	/	/	/	/	/	/	/	/	/	/
Mixed interventions	Oral preparation	Liu 2016	+	+	/	/	/	+	/	/	/	/	/	/	/	/	/	/	/	/	/	/
Mixed interventions	Oral preparation	Chen 2018	+	+	/	+	/	/	/	/	/	/	/	/	/	/	/	/	/	/	/	/
Mixed interventions	Oral preparation	Wang 2019	+	+	+	/	/	/	+	/	/	/	/	/	/	/	/	/	/	/	/	+
Mixed interventions	Oral preparation	Yuan 2019	+	+	/	/	/	+	/	/	/	/	/	/	/	/	/	/	/	/	/	+
Mixed interventions	Oral preparation	Bai 2020	+	+	+	+	/	/	+	/	/	/	/	/	/	/	/	/	+	+	/	+
Mixed interventions	Oral preparation	Xu 2020	+	+	/	/	/	+	/	/	/	+	/	/	/	/	/	/	/	/	/	/
Mixed interventions	Oral preparation	Cai 2022	+	+	+	/	/	+	+	/	/	/	+	/	/	+	+	/	/	/	/	+
Mixed interventions	Oral preparation	Zhu 2022	+	/	/	/	/	/	/	/	/	/	/	/	/	/	/	/	/	/	/	+
Mixed interventions	Oral preparation	Shi 2023	+	+	/	/	/	/	/	/	/	/	/	/	/	/	/	/	/	/	/	+
Mixed interventions	Oral preparation	Zhong 2023	+	+	+	/	/	/	+	/	+	/	/	/	/	/	+	+	+	+	+	+
Mixed interventions	Oral preparation	Li 2024	+	+	+	+	+	/	+	/	/	/	+	/	/	/	/	/	/	/	/	/
Mixed interventions	Oral preparation	Xu 2024	+	+	+	/	/	/	/	/	+	/	/	/	/	/	/	/	/	/	/	+

TCM, traditional Chinese medicine;/,not available.

Clinical symptoms: T: Time to fever resolution; C: Time to resolution of cough; R: Time to resolution of rhinorrhea; N: Time to resolution of nasal congestion; H: Time to resolution of herpes lesions; P: Time to resolution of sore throat; G: Time to resolution of rales; L: Time to resolution of pulmonary rales; S: Time to resolution of sialorrhea; Y: Time to resolution of pharyngeal hyperemia; O: Time to resolution of tonsillar hyperemia; F: Duration of fever; D: Time to resolution of vomiting and diarrhea; K: Time to resolution of sputum production; ADR: Adverse reaction incidence.

Overall, most systematic reviews and meta-analyses suggested that TCM, either alone or in combination with conventional Western medicine, may confer potential benefits in terms of total effective rate and selected short-term symptom outcomes, while some reviews also reported improvements in inflammatory markers. However, the GRADE assessment in the present study was restricted to the outcome of total effective rate, for which the certainty of evidence was predominantly low or very low. Time to fever resolution, duration of cough, and other clinical outcomes were not independently assessed using GRADE. In addition, heterogeneity existed across reviews with respect to disease subtype, intervention regimen, comparator, outcome definition, and assessment criteria. Accordingly, the available efficacy findings should be interpreted with caution, and positive results across different clinical outcomes or intervention strategies should not be assumed to carry an equivalent strength of evidence.

With regard to safety, some included systematic reviews reported the incidence of adverse events, and several also quantitatively pooled relevant safety outcomes. Nevertheless, substantial variation existed in the definitions of adverse events, monitoring methods, severity classification, and duration of follow-up. Moreover, safety outcomes were not independently assessed using GRADE in the present umbrella review. Therefore, the current evidence is insufficient to support definitive conclusions regarding the overall or long-term safety of TCM and integrated traditional Chinese and Western medicine for pediatric AURI.

## 4 Discussion

### 4.1 Main findings

This umbrella review evaluated 41 systematic reviews and meta-analyses of TCM, either alone or in combination with conventional Western medicine, for AURI in children. The existing evidence was comprehensively appraised across multiple dimensions, including reporting completeness, methodological quality, risk of bias, and certainty of evidence specifically for the outcome of total effective rate. Overall, most of the included systematic reviews and meta-analyses suggested that TCM or integrated traditional Chinese and Western medicine may provide potential benefits in terms of total effective rate and selected short-term clinical outcomes. However, our findings also revealed substantial methodological limitations and uncertainty within the current evidence base. Most reviews did not achieve a high level of methodological quality, and the ROBIS assessment indicated a generally high risk of bias. Furthermore, the GRADE assessment of total effective rate showed that the certainty of evidence was predominantly low or very low, with the main reasons for downgrading including methodological limitations of the primary RCTs, inconsistency across studies, imprecision, and potential publication bias.

Accordingly, the potential therapeutic signals observed in the existing systematic reviews cannot yet be translated into high-certainty clinical conclusions. Importantly, the GRADE assessment in the present study was restricted to total effective rate, which is a non-standardized composite outcome; other clinical outcomes, such as time to fever resolution, duration of cough, and adverse events, were not independently evaluated using GRADE. Taken together, the findings regarding methodological quality, risk of bias, and certainty of evidence indicate that the potential clinical benefits of TCM and integrated traditional Chinese and Western medicine for pediatric AURI should continue to be interpreted cautiously.

### 4.2 Major factors limiting the credibility of the evidence

The present review identified varying degrees of methodological limitations and risk of bias among the included systematic reviews and meta-analyses. Notably, AMSTAR 2 classified 37 reviews as having moderate methodological quality, whereas ROBIS judged 38 reviews to be at high risk of bias. Although these findings may appear inconsistent, the two instruments differ substantially in their purposes and evaluative frameworks. AMSTAR 2 primarily assesses the methodological conduct and overall confidence in a systematic review by examining aspects such as protocol development, literature searching, study selection, risk-of-bias assessment of primary studies, statistical synthesis, and interpretation of findings. ROBIS, in contrast, focuses more specifically on whether methodological shortcomings arising during the review process are likely to introduce bias and affect the validity of the review conclusions. Therefore, a systematic review may have a relatively complete methodological structure according to AMSTAR 2 while still being judged at high risk of bias by ROBIS because of deficiencies in study identification and selection, inadequate handling of risk of bias in the primary studies, or potential systematic bias in the synthesis and interpretation of findings. The results obtained with these two tools should therefore be regarded as complementary rather than contradictory, as they reflect different dimensions of the credibility of the existing evidence base.

More specifically, some included reviews lacked protocol registration or did not clearly state whether a complete protocol had been developed before commencement of the review, potentially increasing the risks of selective analysis and selective reporting. The search strategies used in some systematic reviews were also insufficiently comprehensive, with inadequate searches of clinical trial registries, grey literature, conference abstracts, or reference lists. Such limitations may have resulted in the omission of negative findings or unpublished studies and thereby increased the risk of publication bias. In addition, some reviews did not clearly report whether study selection, data extraction, and risk-of-bias assessment were conducted independently by two reviewers, nor did they adequately consider the potential influence of bias in the primary studies when interpreting pooled effect estimates. Although these shortcomings may not necessarily result in a substantial reduction in the overall AMSTAR 2 rating, they may compromise the robustness of evidence synthesis and help explain the high proportion of reviews judged to be at high risk of bias according to ROBIS.

The GRADE assessment further demonstrated that the certainty of evidence for total effective rate was generally low. The principal reasons for downgrading included inadequate reporting of random sequence generation, allocation concealment, and blinding in the primary RCTs, as well as inconsistency between studies, imprecision of effect estimates, and potential publication bias. Taken together, the AMSTAR 2, ROBIS, and GRADE findings suggest that although the current evidence base has some methodological foundation, its conclusions may still be influenced simultaneously by limitations in the quality of the primary studies, weaknesses in the conduct of systematic reviews, and risk of bias. Consequently, positive findings should not be interpreted solely on the basis of statistical significance or apparent consistency across multiple systematic reviews. The potential therapeutic effects of these interventions should be interpreted cautiously, and the available evidence remains insufficient to support strong clinical recommendations.

### 4.3 Limitations of outcome measures and clinical interpretation

The present study found that existing systematic reviews relied heavily on total effective rate as a primary outcome. Although this measure is widely used in clinical research on traditional Chinese medicine in China, its definition is generally based on efficacy criteria established by the individual primary studies. Considerable variation may therefore exist across studies in terms of component events, response thresholds, and assessment time points. Moreover, there is no widely accepted international standard for this outcome, resulting in limited comparability across studies. As a non-standardized composite outcome, total effective rate may reduce the clinical interpretability of effect estimates and limit the comparability and applicability of related findings within the international evidence-based medicine framework (59).

In contrast, clinically meaningful outcomes such as time to fever resolution, time to cough resolution, time to relief of sore throat, and incidence of adverse events were less consistently reported, while evidence relating to inflammatory markers, immune function, and safety outcomes was even more limited. Most cases of pediatric AURI are self-limiting, and symptom trajectories may also be influenced by the natural course of illness, concomitant medications, supportive care, and the timing of outcome assessment (60). Therefore, evaluating the clinical value of an intervention solely on the basis of composite outcomes such as total effective rate has important limitations.

Substantial heterogeneity was also evident in the clinical conditions and interventions covered by the included systematic reviews. The reviews encompassed different clinical presentations of AURI, including the common cold, acute pharyngitis, acute tonsillitis, and herpangina, while the interventions included various Chinese herbal injections, oral Chinese patent medicines, compound herbal formulations, and TCM combined with different conventional Western medical regimens. These clinical conditions may differ in their natural history, symptom profiles, and treatment responses, while the various TCM preparations are not necessarily comparable with respect to composition, dosage form, route of administration, or concomitant treatment.

Accordingly, the overall findings of this umbrella review should primarily be interpreted as a description of the characteristics and credibility of the existing evidence base rather than as evidence that different AURI subtypes or different TCM interventions exert uniform therapeutic effects. Future studies should adopt clearer and more standardized diagnostic criteria and should prospectively conduct stratified or subgroup analyses according to clinical subtype, intervention, and comparator to improve the clinical interpretability of the findings.

### 4.4 Clinical implications

From a clinical perspective, existing systematic reviews suggest that TCM and integrated traditional Chinese and Western medicine may have the potential to improve selected short-term symptom outcomes in children with AURI, including fever, cough, and sore throat. However, these findings are constrained by several factors, including methodological limitations of the primary studies, risk of bias in the systematic reviews, non-standardized outcome measures, and substantial clinical heterogeneity across studies. In addition, the present umbrella review did not independently assess the certainty of evidence for these symptom-related outcomes using GRADE. Therefore, these potential benefits should not currently be interpreted as high-certainty treatment effects or as a basis for strong clinical recommendations.

Clinical application should also take into account the self-limiting nature of most cases of pediatric AURI, as well as patient age, disease severity, previous allergic reactions, concomitant medications, and medication safety. Given the current limitations of the efficacy evidence, the indications for these interventions should not be inappropriately expanded, nor should they be used as substitutes for necessary guideline-concordant diagnosis and treatment.

Particular attention should be paid to safety because several included systematic reviews evaluated Chinese herbal injections in pediatric populations. Although some reviews reported the incidence of adverse events and several quantitatively pooled relevant safety outcomes, substantial variation existed in the definitions of adverse events, monitoring procedures, severity classifications, and duration of follow-up. Moreover, safety outcomes were not independently assessed using GRADE in the present study. Consequently, the available evidence remains insufficient to draw definitive conclusions regarding the overall or long-term safety of Chinese herbal injections or other related interventions in children.

Future studies should prespecify safety outcomes and systematically document general adverse events, serious adverse events, treatment discontinuation due to adverse events, allergic reactions, and infusion-related reactions, with extended follow-up where appropriate. Safety should be evaluated as an independent and clinically important outcome and should undergo formal certainty-of-evidence assessment rather than being treated merely as an ancillary endpoint of efficacy studies. Only when both efficacy and safety are supported by evidence of sufficiently high certainty should these interventions be considered a robust basis for clinical decision-making or guideline recommendations.

### 4.5 Strengths and limitations

A major strength of this study is the systematic inclusion of existing systematic reviews and meta-analyses evaluating TCM and integrated traditional Chinese and Western medicine for pediatric AURI, together with the combined use of PRISMA 2020, AMSTAR 2, ROBIS, and GRADE. This enabled a multidimensional appraisal of the current evidence base in terms of reporting completeness, methodological quality, risk of bias, and certainty of evidence specifically for the outcome of total effective rate. Compared with an individual systematic review, an umbrella review provides a higher-level synthesis of the available evidence and facilitates the identification of common challenges in study design, evidence appraisal, and clinical translation within this field.

Several limitations should also be acknowledged. First, only publicly available systematic reviews and meta-analyses published in Chinese or English were included, which may have introduced language and publication bias. Because clinical research on TCM is predominantly conducted in China, this language restriction is unlikely to have excluded a substantial proportion of the evidence in this field; nevertheless, the potential influence of studies published in other languages cannot be completely ruled out. More importantly, the current evidence base is derived largely from Chinese populations and healthcare settings. Differences across countries and regions in population characteristics, diagnostic and treatment practices, conventional treatment regimens, availability of TCM preparations, and regulatory requirements may limit the generalizability of these findings to non-Chinese populations and other healthcare systems. Future multinational and multicenter studies, together with external validation across diverse healthcare settings, are needed to further assess the generalizability of these interventions.

Second, the same primary RCTs may have been included in multiple systematic reviews and meta-analyses. A complete citation matrix linking primary RCTs to systematic reviews was not constructed, and the corrected covered area (CCA) was not calculated; consequently, the degree of overlap among primary studies could not be quantified. This may have resulted in repeated representation of the same primary study findings across multiple systematic reviews, thereby increasing the apparent weight of certain findings within the overall evidence base and potentially making the consistency of conclusions across reviews appear greater than would be supported by fully independent evidence. Because effect estimates from different systematic reviews were not re-pooled in the present study, study overlap did not generate additional duplicate meta-analytic effect estimates; however, it may still have influenced judgments regarding the volume, consistency, and overall credibility of the evidence. Accordingly, the mere predominance of positive conclusions across systematic reviews was not considered evidence of high credibility, and the review-specific GRADE ratings were not combined into a single cross-review certainty rating. Future umbrella reviews should establish a citation matrix based on complete information from the primary studies and use measures such as the CCA to quantify the extent of study overlap.

Third, this study primarily reassessed evidence that had already been synthesized at the systematic review level and did not independently identify and incorporate the most recent primary RCTs published before the search cutoff but not yet included in existing systematic reviews. Therefore, this umbrella review cannot fully substitute for a direct assessment of the latest primary research evidence.

Fourth, the GRADE assessment was restricted to total effective rate, the most frequently reported outcome across the included systematic reviews, rather than being applied to each systematic review as a whole. Other clinical outcomes, such as time to fever resolution and duration of cough, as well as safety outcomes such as adverse events, were not independently assessed using GRADE. Therefore, the GRADE ratings reported in this study should not be extrapolated to other efficacy or safety outcomes within the same systematic review. In addition, because different systematic reviews may have included overlapping primary RCTs, the 41 GRADE judgments for total effective rate should not be regarded as 41 completely independent bodies of evidence. Their distribution is intended primarily to describe the certainty of evidence for this specific outcome across the existing systematic reviews and should not be used to calculate or infer a single overall GRADE rating across reviews.

Fifth, although some included systematic reviews provided quantitative safety findings, the present study did not uniformly extract or re-synthesize effect estimates, types of adverse events, or severity classifications. Consequently, no quantitative conclusions regarding overall or long-term safety could be drawn. In particular, the safety evidence for Chinese herbal injections in children should be interpreted with caution.

Sixth, this study also has limitations from a virological perspective. Pediatric AURI in the included systematic reviews was primarily diagnosed on the basis of clinical criteria, while standardized laboratory confirmation of causative pathogens was rarely reported. Moreover, robust stratified analyses according to specific respiratory viruses were generally unavailable. Thus, the present findings primarily reflect the overall evidence for children with clinically diagnosed AURI and should not be interpreted as demonstrating definitive efficacy of TCM or integrated traditional Chinese and Western medicine against any specific respiratory virus. Future studies should incorporate standardized etiological testing where feasible and conduct prespecified stratified or subgroup analyses according to pathogen type to improve the specificity and generalizability of treatment-effect estimates.

Finally, assessments using AMSTAR 2, ROBIS, and GRADE inevitably involve a degree of professional judgment. Although all assessments were performed independently by two reviewers, with disagreements resolved through discussion or adjudication by a third reviewer, the potential influence of subjective judgment cannot be entirely eliminated.

### 4.6 Implications for future research

Future research on TCM-related interventions for pediatric AURI should improve methodological rigor at both the primary clinical trial and systematic review levels. Primary RCTs should adhere strictly to CONSORT reporting standards, with appropriate procedures for random sequence generation, allocation concealment, blinding, sample size estimation, and adverse-event reporting. Whenever feasible, multicenter, prospective, and prospectively registered study designs should be adopted (61). With respect to outcome selection, future studies should reduce reliance on non-standardized composite outcomes such as total effective rate and prioritize patient-relevant outcomes that are clinically meaningful, reproducible, and internationally comparable. These may include overall symptom duration, duration of fever, time to relief of cough and sore throat, antibiotic use, revisit or reconsultation rates, adverse events, and health-related quality of life. The development of a core outcome set specifically for studies of TCM interventions in pediatric AURI should also be encouraged (62).

At the systematic review level, greater emphasis should be placed on prospective protocol registration, comprehensive searches of bibliographic databases, clinical trial registries, and grey literature, and standardized procedures involving independent study selection and data extraction by two reviewers. Risk of bias in primary studies, between-study inconsistency, and publication bias should be thoroughly assessed and explicitly incorporated into the interpretation of findings. GRADE assessments should be conducted at the level of individual outcomes and their corresponding bodies of evidence, with transparent reporting of each downgrading domain and the final certainty rating. The GRADE rating for a single outcome should not be extrapolated to an entire systematic review or to other clinical outcomes. For umbrella reviews, overlap of primary RCTs across systematic reviews should also be carefully addressed. Where sufficient data are available, a citation matrix should be constructed and quantitative measures such as the corrected covered area should be used to assess the extent of overlap.

Safety evidence warrants particular attention in pediatric populations. Future studies should prespecify safety outcomes as important study endpoints and systematically report general adverse events, serious adverse events, treatment discontinuation due to adverse events, allergic reactions, and infusion-related reactions. Interventions requiring particular caution, including Chinese herbal injections, should be evaluated with more comprehensive safety monitoring and, where appropriate, longer follow-up. Only through further standardization of study design, outcome selection, certainty-of-evidence assessment, and safety reporting can the true clinical value of TCM and integrated traditional Chinese and Western medicine for pediatric AURI be determined with greater confidence.

## 5 Conclusions

Existing systematic reviews and meta-analyses suggest that TCM and integrated traditional Chinese and Western medicine may provide potential benefits for children with AURI in terms of total effective rate and selected short-term clinical outcomes. However, the included systematic reviews were generally characterized by a high risk of bias, and the GRADE ratings for total effective rate, a non-standardized composite outcome, were predominantly low or very low. Because other efficacy and safety outcomes, including time to fever resolution, duration of cough, and adverse events, were not independently assessed using GRADE in the present study, these certainty ratings should not be extrapolated to all clinical outcomes.

In addition, methodological limitations of the systematic reviews, potential overlap of primary RCTs across reviews, clinical heterogeneity in disease subtypes and interventions, and the non-standardized nature of total effective rate further constrain the credibility and clinical interpretability of the current evidence. Accordingly, the available evidence is insufficient to support strong clinical recommendations, and existing findings should be interpreted cautiously. Future research should prioritize rigorously designed primary RCTs, standardized and clearly patient-relevant outcome measures, and high-quality systematic reviews to further establish the efficacy and safety of these interventions.

## Data Availability

The original contributions presented in the study are included in the article/supplementary material, further inquiries can be directed to the corresponding author/s.

## References

[1] Chonmaitree T Revai K Grady JJ Clos A Patel JA Nair S. Viral upper respiratory tract infection and otitis media complication in young children. Clin Infect Dis. (2008) 46(6):815–23. 10.1086/528685 18279042 PMC2744371

[2] Li Y Fu X Ma J Zhang J Hu Y Dong W. Altered respiratory virome and serum cytokine profile associated with recurrent respiratory tract infections in children. Nat Commun. (2019) 10(1):2288. 10.1038/s41467-019-10294-x 31123265 PMC6533328

[3] Li Z-J Zhang H-Y Ren L-L Lu Q-B Ren X Zhang C-H. Etiological and epidemiological features of acute respiratory infections in China. Nat Commun. (2021) 12(1):5026. 10.1038/s41467-021-25120-6 34408158 PMC8373954

[4] Butler AM Brown DS Durkin MJ Sahrmann JM Nickel KB O’Neil CA. Association of inappropriate outpatient pediatric antibiotic prescriptions with adverse drug events and health care expenditures. JAMA Netw Open. (2022) 5(5):e2214153. 10.1001/jamanetworkopen.2022.14153 35616940 PMC9136626

[5] Ping R Li L Xilian Z Quanhui L Huimin Y Deli X. Clinical practices s of traditional Chinese medicine for acute upper respiratory tract infection in children. J Tradit Chin Med. (2018) 38(4):504–10. 10.1016/S0254-6272(18)30882-3 32186075

[6] Chen Z Ye SY. Research progress on antiviral constituents in traditional Chinese medicines and their mechanisms of action. Pharm Biol. (2022) 60(1):1063–76. 10.1080/13880209.2022.2074053 35634712 PMC9154771

[7] Cortese S Adamo N Del Giovane C Mohr-Jensen C Hayes AJ Carucci S. Comparative efficacy and tolerability of medications for attention-deficit hyperactivity disorder in children, adolescents, and adults: a systematic review and network meta-analysis. Lancet Psychiatry. (2018) 5(9):727–38. 10.1016/S2215-0366(18)30269-4 30097390 PMC6109107

[8] Lunny C Kanji S Thabet P Haidich AB Bougioukas KI Pieper D. Assessing the methodological quality and risk of bias of systematic reviews: primer for authors of overviews of systematic reviews. BMJ Med. (2024) 3(1):e000604. 10.1136/bmjmed-2023-000604 38826514 PMC11141200

[9] Page MJ McKenzie JE Bossuyt PM Boutron I Hoffmann TC Mulrow CD. The PRISMA 2020 statement: an updated guideline for reporting systematic reviews. Br Med J. (2021) 372:n71. 10.1136/bmj.n71 PMC8005924 33782057

[10] Jiang ZF Shen KL Shen Y. Zhu Futang Textbook of Pediatrics. 8th edn. Beijing: People’s Medical Publishing House (2015).

[11] Huang GY Wang TY. Pediatrics. Beijing: People’s Medical Publishing House (2022). p. 997.

[12] Lorenz RC Matthias K Pieper D Wegewitz U Morche J Nocon M. A psychometric study found AMSTAR 2 to be a valid and moderately reliable appraisal tool. J Clin Epidemiol. (2019) 114:133–40. 10.1016/j.jclinepi.2019.05.028 31152864

[13] Gates M Gates A Guitard S Pollock M Hartling L. Guidance for overviews of reviews continues to accumulate, but important challenges remain: a scoping review. Syst Rev. (2020) 9(1):254. 10.1186/s13643-020-01509-0 33148319 PMC7643411

[14] Whiting P Savović J Higgins JPT Caldwell DM Reeves BC Shea B. ROBIS: a new tool to assess risk of bias in systematic reviews was developed. J Clin Epidemiol. (2016) 69:225–34. 10.1016/j.jclinepi.2015.06.005 26092286 PMC4687950

[15] Neuenschwander M Ballon A Weber KS Norat T Aune D Schwingshackl L. Role of diet in type 2 diabetes incidence: umbrella review of meta-analyses of prospective observational studies. Br Med J. (2019) 366:l2368. 10.1136/bmj.l2368 PMC6607211 31270064

[16] Guyatt GH Oxman AD Vist GE Kunz R Falck-Ytter Y Alonso-Coello P. GRADE: an emerging consensus on rating quality of evidence and strength of recommendations. Br Med J. (2008) 336(7650):924–6. 10.1136/bmj.39489.470347.AD PMC2335261 18436948

[17] Siemieniuk RAC Bartoszko JJ Zeraatkar D Kum E Qasim A Díaz Martinez JP. Drug treatments for COVID-19: living systematic review and network meta-analysis. Br Med J. (2020) 370:m2980. 10.1136/bmj.m2980 PMC7390912 32732190

[18] Li Y Wei WQ. Meta-analysis of the efficacy of reduning in the treatment of upper respiratory tract infection. Chin J Pharmacoepidemiol. (2010) 19(6):314–9.

[19] Tao JF Zhang T Shi XY Zhu J. Meta-analysis of reduning and xiyanping injections for acute upper respiratory tract infection. West J Tradit Chin Med. (2015) 28(2):63–6.

[20] Guo ZL Su ZN Wang ZF Luo XM. Meta-analysis of reduning versus ribavirin in the treatment of pediatric acute upper respiratory tract infection. Chin Tradit Pat Med. (2016) 38(2):278–83.

[21] Wu GH Chen FC Li P. Meta-analysis of the efficacy of yanhuning versus ribavirin in the treatment of herpangina in children. Mod J Integr Tradit Chin West Med. (2013) 22(8):821–3.

[22] Shi XY Zhou T Chen FC Fang BX Li P. Xiyanping versus ribavirin aerosol inhalation for respiratory tract infections in children: a meta-analysis of randomized controlled trials. Int J Tradit Chin Med. (2014) 11:974–7.

[23] Lian BT Wu ZL Li ZZ Chen JC Hu XY. Meta-analysis of xiyanping versus ribavirin for pediatric herpangina. China Pharmacy. (2016) 27(3):337–40.

[24] Ren XL Li AP Li XH Wang YP Wang Q Zhong ML. Meta-analysis of the efficacy of xiyanping injection versus ribavirin in the treatment of pediatric acute upper respiratory tract infection. Chin Tradit Pat Med. (2017) 39(3):480–5. 10.3969/j.issn.1001-1528.2017.03.008

[25] Liu SY Chen Y Guo HH Wang R Liu FY Cheng GH. Systematic review of the efficacy and safety of xiyanping injection for pediatric upper respiratory tract infection. Pharm Clin Chin Mater Med. (2017) 33(3):165–71.

[26] Han YH Zhang H. Systematic review of Tanreqing Injection for pediatric upper respiratory tract infection. Chin J Exp Tradit Med Formulae. (2016) 22(12):215–9.

[27] Liu ZQ Chen CL Wang BL Qin HX Yang SC. Systematic review of two heat-clearing and detoxifying injections for pediatric upper respiratory tract infection. Asia Pac Tradit Med. (2017) 13(23):138–42.

[28] Zhang J Guo HJ Shi N Fan YX Wang H Liu CY. Meta-analysis of the effectiveness of lanqin oral liquid in the treatment of pediatric upper respiratory tract infection. Beijing J Tradit Chin Med. (2015) 34(11):863–6.

[29] Yuan D Xie HH Wang SC Yuan B Chen M. Meta-analysis of the clinical efficacy of xiaoer chiqiao qingre granules for acute upper respiratory tract infection. China J Tradit Chin Med Pharm. (2016) 31(1):89–92.

[30] Zhong CL Hu SY Li JF Wu JT Xu Q. Meta-analysis of jinlian qingre effervescent tablets for pediatric acute upper respiratory tract infection. Drug Eval Res. (2017) 40(3):393–9.

[31] Hu Q Sun T-T Hong J Fang J-Y Xiong H Meltzer SJ. Andrographis paniculata (Chuān Xīn Lián) for symptomatic relief of acute respiratory tract infections in adults and children: a systematic review and meta-analysis. PLoS One. (2017) 12(8):e0181780. 10.1371/journal.pone.0169691 28783743 PMC5544222

[32] Ma YN Hu SY Qiu YP Zhang CC Li CL Jin Y. Meta-analysis of xiaoer chiqiao qingre granules versus ribavirin for pediatric acute upper respiratory tract infection. Asia Pac Tradit Med. (2020) 16(3):177–81.

[33] Fang BX Chen FC Wu SC Shi XY Feng LB Qian N. Meta-analysis of reduning injection versus ribavirin for herpangina in children. Guiding J Tradit Chin Med Pharm. (2012) 18(9):99–102.

[34] Xu JL Liang ZR Guo ZL Li ZC Deng LM. Meta-analysis of xiyanping combined with ribavirin for pediatric herpangina. Chin J Exp Tradit Med Formulae. (2016) 22(4):195–200.

[35] Li L Xie Y Chai Y Liao X Pei W. Qingkailing injection for uncomplicated upper respiratory tract infection: a systematic review and meta-analysis. Eur J Integr Med. (2016) 8(5):609–18. 10.1016/j.eujim.2016.06.019

[36] Peng J Tan BY Ning N. Systematic review of the efficacy and safety of Yanhuning Injection versus interferon-α for pediatric upper respiratory tract infection. J Hunan Univ Chin Med. (2019) 39(8):1035–9.

[37] Luo M Hua P Tang Y. Systematic review of Ganke Shuangqing Capsules for acute upper respiratory tract infection. China Pharmacy. (2011) 22(15):1419–22.

[38] Chen LJ Tang R Liu LL Hu ZB. Systematic review of xiaoer chiqiao qingre granules for pediatric upper respiratory tract infection. China Pharmaceuticals. (2013) 22(14):47–50.

[39] Tao H Ma R Zeng XT Yin QF Li YQ Li BH. Meta-analysis of the safety and efficacy of kaihoujian spray combined with conventional therapy for herpetic oral diseases in children. Med J West China. (2017) 29(11):1540–6.

[40] Rong P Zhang XL Liu QH Yan HH Pan GY. Meta-analysis of the clinical efficacy of an’erning granules for respiratory infections in children. Liaoning J Tradit Chin Med. (2021) 48(2):14–9.

[41] Zhang Y Xia R Liang S Hu X Dai M Li Y. Chinese patent herbal medicine (Shufeng Jiedu Capsule) for acute upper respiratory tract infections: a systematic review and meta-analysis. Integr Med Res. (2021) 10(3):100726. 10.1016/j.imr.2021.100726 33996460 PMC8099504

[42] Wang SH Zhang FX Zhu TY Wu HF Qin PJ. Meta-analysis of shufeng jiedu capsule combined with western medicine for pediatric acute upper respiratory tract infection. World Chin Med. (2023) 18(22):3211–7.

[43] Chen LJ Liu LL Hu ZB Lu HB. Systematic review of xiyanping combined with conventional treatment for pediatric herpangina. China Pharmaceutics. (2012) 21(15):19–23.

[44] Guo ZL Su ZN. Meta-analysis of yanhuning in the treatment of pediatric acute upper respiratory tract infection. J Liaoning Univ Tradit Chin Med. (2016) 18(2):83–7.

[45] Qiu JL Xu H Niu MM Chen D Li Y. Meta-analysis of randomized controlled trials of traditional Chinese medicine for fever in pediatric upper respiratory tract infection. J Nanjing Univ Tradit Chin Med. (2014) 30(1):15–8.

[46] Liu LJ Zhang X Qu WY Jing WC Wang YP. Meta-analysis of traditional Chinese medicine in the treatment of herpangina in children. Guiding J Tradit Chin Med Pharm. (2016) 22(22):88–91.

[47] Duan LF Weng XH. Systematic review of the efficacy and safety of Lanqin Oral Liquid for upper respiratory tract infection in children. Chin J Mod Drug Appl. (2016) 10(9):3–5.

[48] Yuan B Sun YL Wu YW Xu SJ. Meta-analysis of the efficacy and safety of lanqin oral liquid for herpangina in children. China Pharmacy. (2019) 30(12):1702–6.

[49] Chen X Yu Y Li SN Yun QL Wu B Qian LQ. Meta-analysis of the clinical efficacy and safety of ribavirin alone versus combined with xiaoer chaigui tuire granules for pediatric acute upper respiratory tract infection. Infect Inflamm Repair. (2018) 19(2):72–82.

[50] Li Y Chang WJ Zhang XD Zhang JW Chen W. Systematic review and meta-analysis of the efficacy and safety of xiaoer chaigui tuire granules for acute upper respiratory tract infection. Chin J Gen Pract. (2024) 22(11):1957–62.

[51] Wang SH Liu JF Qin PJ Gao B. Meta-analysis of shuanghuanglian oral liquid alone or combined with conventional therapy for pediatric acute upper respiratory tract infection. Res Pract Chin Med. (2019) 33(6):63–8.

[52] Bai Y Li YX Shi YJ. Meta-analysis of the clinical efficacy and safety of Pudilan Xiaoyan Oral Liquid for pediatric upper respiratory tract infection. China J Chin Mater Med. (2020) 45(9):2203–9. 10.19540/j.cnki.cjcmm.20200102.501 32495572

[53] Xu Y Yan YB. Meta-analysis of Pudilan Xiaoyan Oral Liquid for pediatric herpangina. China’s Naturopathy. (2020) 28(22):52–5.

[54] Cai LL Xu YQ Hua ML Hu SY. Systematic review and meta-analysis of jian’er qingjie liquid for upper respiratory tract infection in children. Chin J Ethnomed Ethnopharm. (2022) 31(18):99–106.

[55] Zhu YP Ma T. Meta-analysis of the clinical efficacy of Qinxiang Qingjie Oral Liquid for upper respiratory tract infection in children. Electron J Clin Med Lit. (2022) 9(17):33–6.

[56] Shi Y Wu LQ Zhang Y Zhang YQ. Meta-analysis of the efficacy and safety of oral yinqiao powder for pediatric upper respiratory tract infection. Chin J Ethnomed Ethnopharm. (2023) 32(20):101–7.

[57] Zhong N Gong WH Wan T. Meta-analysis and trial sequential analysis of Chaihuang Granules in the treatment of pediatric upper respiratory tract infection. China J Chin Mater Med. (2023) 48(19):5377–88. 10.19540/j.cnki.cjcmm.20230605.501 38114127

[58] Xu LC Han QX Pan J Zhou PD Wei JY Zhu WT. Meta-analysis of the efficacy and safety of kanggan granules for pediatric upper respiratory tract infection. China J Pharm Econ. (2024) 19(4):43–51.

[59] Cordoba G Schwartz L Woloshin S Bae H Gøtzsche PC. Definition, reporting, and interpretation of composite outcomes in clinical trials: systematic review. Br Med J. (2010) 341:c3920. 10.1136/bmj.c3920 PMC2923692 20719825

[60] Van Driel ML Scheire S Deckx L Gevaert P De Sutter A. What treatments are effective for common cold in adults and children? Br Med J. (2018) 363:k3786. 10.1136/bmj.k3786 30305295

[61] Schulz KF Altman DG Moher D. CONSORT 2010 statement: updated guidelines for reporting parallel group randomised trials. J Pharmacol Pharmacother. (2010) 1(2):100–7. 10.4103/0976-500X.72352 21350618 PMC3043330

[62] Kirkham JJ Gorst S Altman DG Blazeby JM Clarke M Devane D. Core outcome set-STAndards for reporting: the COS-STAR statement. PLoS Med. (2016) 13(10):e1002148. 10.1371/journal.pmed.1002148 27755541 PMC5068732

